# Senescence as a trade-off between successful land colonisation and longevity: critical review and analysis of a hypothesis

**DOI:** 10.7717/peerj.12286

**Published:** 2021-11-02

**Authors:** Tomasz Bilinski, Aneta Bylak, Krzysztof Kukuła, Renata Zadrag-Tecza

**Affiliations:** 1 Department of Biochemistry and Cell Biology, Faculty of Biology and Agriculture, University of Rzeszów, Rzeszów, Poland; 2 Department of Ecology and Environmental Protection; Institute of Agricultural Sciences, Land Management and Environmental Protection, University of Rzeszów, Rzeszów, Poland; 3 Department of Biochemistry and Cell Biology, Institute of Biology and Biotechnology, University of Rzeszów, Rzeszów, Poland

**Keywords:** Senescence, Aging, Longevity, Energy, Life program, Growth, Regeneration

## Abstract

**Background:**

Most common terrestrial animal clades exhibit senescence, suggesting strong adaptive value of this trait. However, there is little support for senescence correlated with specific adaptations. Nevertheless, insects, mammals, and birds, which are the most common terrestrial animal clades that show symptoms of senescence, evolved from clades that predominantly did not show symptoms of senescence. Thus, we aimed to examine senescence in the context of the ecology and life histories of the main clades of animals, including humans, and to formulate hypotheses to explain the causes and origin of senescence in the major clades of terrestrial animals.

**Methodology:**

We reviewed literature from 1950 to 2020 concerning life expectancy, the existence of senescence, and the adaptive characteristics of the major groups of animals. We then proposed a relationship between senescence and environmental factors, considering the biology of these groups of animals. We constructed a model showing the phylogenetic relationships between animal clades in the context of the major stages of evolution, distinguishing between senescent and biologically ‘immortal’ clades of animals. Finally, we synthesised current data on senescence with the most important concepts and theories explaining the origin and mechanisms of senescence. Although this categorisation into different senescent phenotypes may be simplistic, we used this to propose a framework for understanding senescence.

**Results:**

We found that terrestrial mammals, insects, and birds show senescence, even though they likely evolved from non-senescent ancestors. Moreover, secondarily aquatic animals show lower rate of senescence than their terrestrial counterparts. Based on the possible life histories of these groups and the analysis of the most important factors affecting the transition from a non-senescent to senescent phenotype, we conclude that aging has evolved, not as a direct effect, but as a correlated response of selection on developmental strategies, and that this occurred separately within each clade. Adoption of specific life history strategies could thus have far-reaching effects in terms of senescence and lifespan.

**Conclusions:**

Our analysis strongly suggests that senescence may have emerged as a side effect of the evolution of adaptive features that allowed the colonisation of land. Senescence in mammals may be a compromise between land colonisation and longevity. This hypothesis, is supported by palaeobiological and ecological evidence. We hope that the development of new research methodologies and the availability of more data could be used to test this hypothesis and shed greater light on the evolution of senescence.

## Introduction

Organisms develop several adaptive mechanisms in response to selection pressures in the environment. These include morphological adaptations that enable locomotion or foraging (Gordon et al., 2017; McGraw, Pampush & Daegling, 2012), physiological adaptations such as the ability to hibernate (Ruf & Bieber, 2020), and behavioural adaptations such as predator avoidance and migration during adverse climatic conditions (Wong & Candolin, 2014). Many of these adaptations have been rigorously characterised in commonly studied organisms. For instance, the adaptation of aquatic animals to life on land and the accompanying evolutionary changes involved in this transition have been well studied (Takei, 2015). Traits that increase the survival and reproduction of organisms in a particular environment are considered adaptations. While all life forms on Earth are phylogenetically related, they exhibit remarkable diversity due to different natural selection pressures, whereby some traits were favoured by the environment over others (Darwin, 1859).

Animals are restricted by features of their environment in terms of their functions such as locomotion. Locomotion on land presents different challenges than that in water, with low friction being replaced by the stronger effects of gravity (Charig, 1972). Moreover, the environment may impose reproductive and/or survival costs on animals (*e.g.*, related to the maintenance a constant temperature), affecting their activity, metabolism, and life expectancy. Therefore, animals on land may differ both in terms of senescence phenotype and their maximal lifespan (MLS) values from animals in water.

Some clades of animals living on land are represented by only a few species that have MLS of more than 100 years, while such species have been described much more frequently in the aquatic environment, especially among fish (Carey & Judge, 2000). Moreover, several animal species with high regenerative potential and indeterminate growth have also been reported (Bernard, Compagnoni & Salguero-Gómez, 2019). Although all animals decline in condition with age (Finch, 1990; Jones et al., 2014; Vaupel et al., 2004), symptoms of senescence such as progressive loss of function, decreased fertility, and increased risk of mortality with age (Medawar, 1952) are not always visible (Bilinski, Bylak & Zadrag-Tecza, 2016).

Several unresolved questions remain in our understanding of the process of senescence, including whether it is inevitable and universal among living organisms (Blagosklonny, 2006; Blagosklonny, 2012; Cohen, 2018; Flatt & Partridge, 2018; Jones & Vaupel, 2017; Kirkwood & Austad, 2000). The inevitability of senescence is largely based on the mechanistic theory of wear developed by Max Rubner, who pointed to metabolic rate as a factor causing variability in the lifespan of organisms. Rubner, based on the analysis of several animal species, showed that larger animals have a lower metabolic rate compared to small animals and they live longer, so slower metabolism may be associated with longevity (Rubner, 1908). Overall, when analysing different groups of animals, we should be aware of the potential bias generated by variation in size (Speakman, 2005). The conclusion that total metabolic energy consumption per life is constant then became the basis for Raymond Pearl’s ‘rate of living’ theory (Pearl, 1928). Energy metabolism and its role in lifespan is an interesting but unresolved issue, therefore it is still under discussion in the literature (Speakman et al., 2002; Speakman, 2005). The theory of accumulation of toxic by-products of metabolism (Harman, 1956; Harman, 2009) may also be derived from this concept, considering that energy metabolism generates free radicals which can damage macromolecules.

The question then arises if this decline occurs, why doesn’t natural selection get rid of it? Senescence requires an ultimate (evolutionary) explanation. Causes of senescence have been studied by evolutionary biologists in a wide range of species (Flatt & Partridge, 2018; Maklakov & Chapman, 2019). According to the theory of antagonistic pleiotropy formulated by Williams, senescence can be treated as a consequence of the positive selection of genes that show benefits for a young organism but have negative effects on an older one (Williams, 1957). Similarly, the “disposable soma” theory for the evolution of senescence developed by Kirkwood suggests that there are specific trade-offs in the allocation of limiting resources between self-maintenance and other activities, mainly reproduction (Kirkwood, 1977). According to Kirkwood, senescence is the result of unrepaired damage that accumulates with age, because the cost of the mechanisms that prevent or repair these damages might be balanced by the benefits due to investment of energy in reproduction (Kirkwood, 1977; Kirkwood & Holliday, 1979). Hence, both these theories proposed by Williams and Kirkwood assume specific optimisation of senescence, by the existence of trade-offs between fecundity and longevity, or between longevity and physiological functions, but maintain its inevitability.

The inevitability of senescence is associated with the assumption that the mortality rates of all organisms should increase with age. However, several multicellular organisms show diverse life history strategies with distinct mortality and fertility trajectories (Capdevila et al., 2020; Jones et al., 2014). These strategies may include senescence—increase mortality with age, negligible senescence—mortality remains roughly constant, or negative senescence—decline in mortality with age after reproductive maturity. Negligible senescence can be observed in organisms, such as the American lobster (*Homarus americanus*), rockfish (*Sebastes spp.*), and tortoises (*Testudinidae*) (Finch, 1990; Finch, 1998) that show very little or no increase in their mortality rate with age. In contrast, a decline in mortality rate may be favoured in cases of negative senescence as proposed by Vaupel et al. (2004). Species that continue to grow and maintain reproductive capacity after maturity, such as teleost fish and many plant species may exhibit negligible or negative senescence (Vaupel et al., 2004).

Senescence can be considered in the context of the distinction between soma and germ line and connected with them terminal differentiation in sexual reproduction. Species or developmental stages of animal species that specialise in sexual reproduction are generally mortal and often senescent. Life of these individuals starts from a single cell of the germ line, whereas their body does not take a direct part in creation of the progeny and undergo senescence (Bilinski, Bylak & Zadrag-Tecza, 2016; Sawada, Inoue & Iwano, 2014). In turn, agametically reproducing animals, in a biological sense, can be treated as “biologically immortal”, if we ignore the risk of mortality arising from external factors (Devarapalli et al., 2014; Reitzel, Stefanik & Finnerty, 2011; Skold & Obst, 2011). These species do not have a distinct soma, therefore the body of the initial organism directly participates in the formation of the progeny (Bilinski, Bylak & Zadrag-Tecza, 2016). However, this pattern is not consistently observed in other kingdoms of organisms. For instance, bacteria have been shown to be senescent (Ackermann, Stearns & Jenal, 2003; Stewart et al., 2005) and plants exhibit evidence of senescence in their asexual reproduction phase (Baudisch et al., 2013; Baudisch & Vaupel, 2012; Mencuccini & Munné-Bosch, 2017). Furthermore, an important caveat must be made with respect to the determination of the level in the system at which the analysed unit is located. For instance, analysis at the level of gametes shows that the latter are in fact “immortal,” unlike the individual “vehicles” or bodies that carry these gametes (Dawkins, 1982).

In some cases, it is difficult unambiguously separate the external and internal forces driving senescent declines in mortality in wild populations. Some organisms may be intrinsically “immortal” by nature, but they may still be damaged by their environment. Thus, these “biologically immortal” organisms may die but they do not senesce. Animals die from a variety of causes, and external mortality plays a large role in wild populations (Nussey et al., 2008; Promislow, 1991).

Considering a wide diversity of life patterns including senescence, negligible senescence and non-senescence with a corresponding long or short lifespan, the current evolutionary theories cannot completely explain the mechanisms of life history evolution in clades with various senescence phenotypes, but they may offer insights for further research. Bilinski and co-authors indicated that senescence might not be as common as expected among animals (Bilinski, Bylak & Zadrag-Tecza, 2016; Bilinski, Paszkiewicz & Zadrag-Tecza, 2015). Senescence occurs mainly in those species that lose their ability to grow upon reaching sexual maturity (Ricklefs, 2010). This involves total (*e.g.*, insects) or partial (*e.g.*, birds and mammals) loss of regenerative capacity or ability to repair damage that occurs after sexual maturity (Galis, Wagner & Jockusch, 2003; Rolff, Johnston & Reynolds, 2019).

Senescence and lifespan can be considered either independently or as interdependent factors. Different organisms vary significantly in their lifespan. Literature offers examples of cases where lifespan is used as a measure of intensity of senescence, which may suggest that lifespan variability is caused mainly by the rate of senescence. However, studies carried out by Peron et al. (2019) on a broad range of mammal species have shown that variability of mortality increase with age accounts for less than half of the variance in lifespan and is therefore inadequate for analysis of senescence. In turn, Baudisch (2011) proposes that to compare the senescence among different species, it is more appropriate to use factors such as pace (the time-scale on which mortality progresses) and shape of senescence (does not depend on time) which express changes of mortality with age. Nevertheless, understanding the link between these parameters requires an understanding of the environmental conditions of various species and their life strategies. As highlighted by Cohen (2018), senescence is not regulated by one universal mechanism but there are the confluences of a large number of mechanisms, which differ also among related species. Therefore, the analysis both of senescence and lifespan in the context of adaptation to aquatic or land environment, may help investigate the evolutionary circumstances shaping lifespan or determine the extent to which senescence correlates with lifespan across species. Large differences in lifespan observed among animals living in different environments are partly due to the implementation of specific life programs or life strategies (Bilinski, Bylak & Zadrag-Tecza, 2016). Existing theories for the evolution of senescence do not exhaustively account for patterns of senescence.

In this review, we proposed a novel hypothesis on the evolution of senescence. Our work contributes to our understanding of the evolution of senescence. The aim of the current study was to present senescence in the context of primary ecological aspects and life histories of the main clades of animals, including humans. In addition, the aim was to formulate hypotheses to explain the causes of senescence phenotype and their origin in the main clades of terrestrial animals.

### Survey methodology

The analysis included in this review was carried out on the basis of kingdom *Animalia*. To avoid bias in the selection of species, we referred to comprehensive studies that summarised life expectancy patterns across animal clades. We obtained data on vertebrates from a study by Carey & Judge (2000). Data on the life expectancy of invertebrates were obtained from multiple reviews (Giraldo et al., 2016; Wanninger, 2015; Ward, 1992). Additionally, we used data from AnAge, which is a comprehensive database of animal longevity records and related life-history traits (available online: http://genomics.senescence.info/species/). AnAge was compiled from the literature and large-scale datasets (De Magalhaes & Costa, 2009), and currently features 4,244 entries. Data on maximal lifespan available at https://www.demogr.mpg.de/longevityrecords/ were also used. The records of the Maximum Lifespan (MLS) values of animal species belonging to the main clades were verified against previous studies and used to construct a database for further analyses. We split MLSs into three classes: MLS<5 years (short), 5 ≤MLS<35 years (intermediate), and MLS ≥35 years (long). Each clade was assigned to an MLS category based on their predominant MLS phenotypes (Table S1). This maximum lifespan categorisations into three classes is arbitrary.

**Figure 1:**
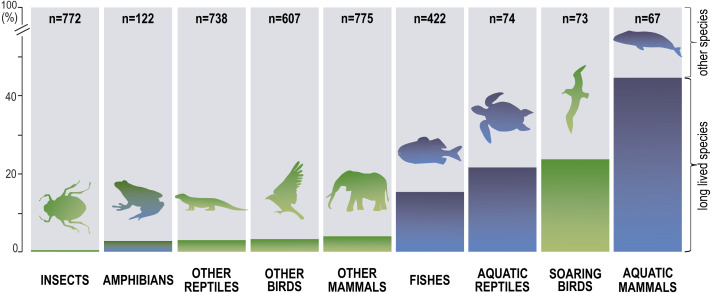
Percentage share of species with long maximal lifespan (≥ 35 years) among all analysed species.

**Figure 2:**
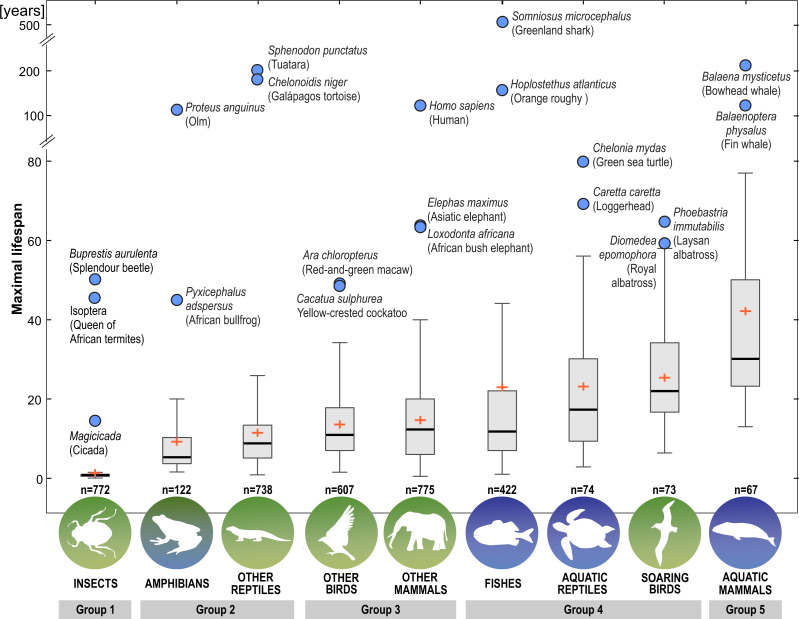
Differences among animal groups in terms of maximal lifespans. Non-parametric ANOVA (Kruskal–Wallis test; H_8,36_ = 1963.25; *P* < 0.00001) indicates significant differences among animal groups in terms of maximal lifespans. Groups 1–5 were identified based on Dunn’s post-hoc test. Boxes show interquartile range. Median value is indicated by horizontal line and mean value is indicated by cross. Whiskers indicate non-outlier range. Circles indicate longevity records.

**Figure 3:**
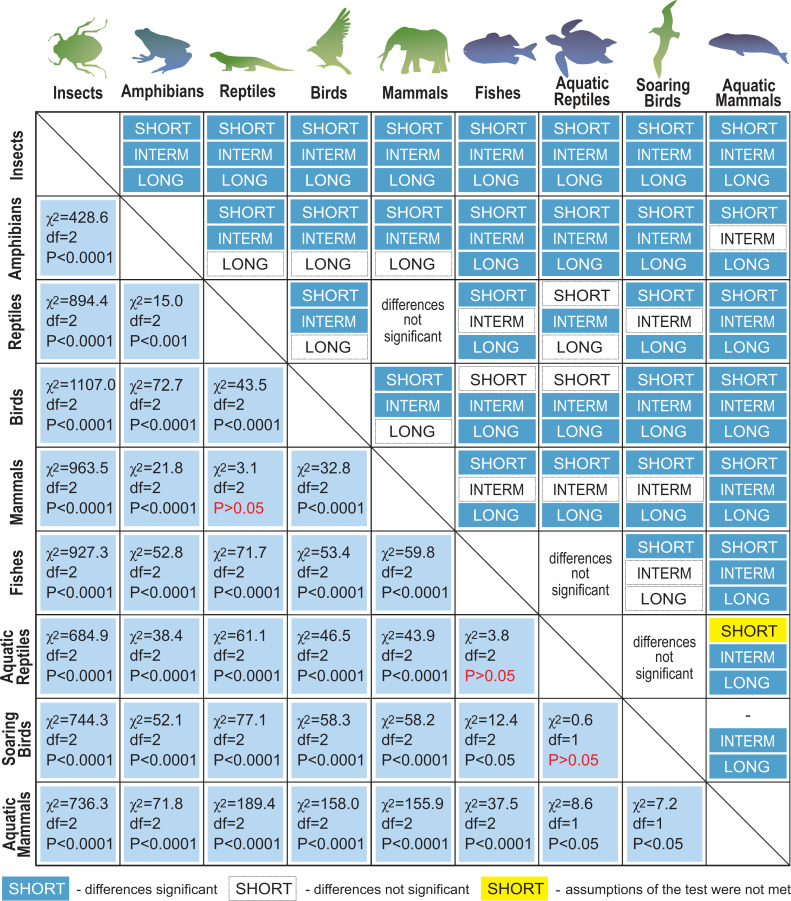
Results of maximal lifespan (MLS) categories comparisons, performed separately for each pair of animal groups in thirty-six 2 ×3 contingency tables from which the chi-squared values (*χ*2) were calculated. MLSs classes: SHORT, MLS <5 years; INTERM –INTERMEDIATE, 5 ≤ MLS <35 years; LONG, MLS ≥ 35 years.

Next, we distinguished subgroups within the clades containing secondary aquatic species. Accordingly, mammals were divided into aquatic mammals and other mammals, and reptiles were divided into aquatic reptiles and other reptiles. In order to take into account possible differences between typical soaring birds with reduced energy costs of flight and more land-bound birds, we also created subgroups of soaring birds and other birds. The percentage of long-living species among all analysed species in these different groups of animals is shown in Fig. 1. Studies of evolutionary correlations commonly use phylogenetic regression (*i.e.,* phylogenetic generalized least squares) to assess trait covariation in a phylogenetic context. However, there are opinion that while this approach is appropriate for evaluating trends in one or a few traits, it is incapable of assessing patterns in highly multivariate data, as the large number of variables relative to sample size prohibits parametric test statistics from being computed (Adams, 2014). In this article, we use a statistical procedure for performing non-parametric ANOVA (Kruskal-Wallis test), that can accommodate our datasets. Average MLS was compared across animal groups using the non-parametric ANOVA and Dunn’s post-hoc tests (Fig. 2). MLS effects (MLS categories) were compared separately for each pair of animal clades in thirty six 2 ×3 contingency tables from which chi square values were calculated (Zar, 2010; Fig. 3).

We reviewed literature (1950–2020) on the life expectancy, the existence of aging symptoms in animals, the described causes of death, as well as the adaptive characteristics of the main groups of animals. We searched the following publishers and databases: Elsevier’s ScienceDirect, PubMed, Springer’s SpringerLink, Wiley Online Library, Google Scholar, and NCBI Bookshelf (Table S2).

Publications where the searched term sets were found in the title, keywords, or abstract, met the inclusion criteria, and these articles were then analysed at the full-text level. All relevant studies that met the search criteria were included in this review. Studies using the same data from various publications were eliminated to avoid redundancy.

Three sets of words were used in the literature search. The senescence set consisted of words related to senescence, and included ageing, aging, damage, death, decrease in fertility, life, life program, life-span, lifespan, longevity, loss of function, mortality, non-senescence, repair, regeneration, rejuvenation, and senescence. The animal features set consisted of words related to the adaptive features of animals, including ability, activity, adaptation, agametic, body size, breathing, burrowing, development, clonal, colonial, ecology, extinction, environment, evolution, factor, feature, fecundity, flight, flying, gas exchange, growth, land, locomotion, maturity, metamorphosis, offspring, oxygen, physiology, process, reproduction, sexual, strategy, survival, swimming, temperature, terrestrial, walking, and water. The animal clades set consisted of the names of clades and groups of animals, and included animals, amphibians, annelids, birds, chordates, cnidarians, crustaceans, dinosaurs, echinoderms, fish, gastropods, hexapods, insects, invertebrates, jawed fish, jawless fish, lancelets, mammals, metazoans, molluscs, myriapods, porifers, ribbon-worms, reptiles, sauropsids, slugs, snails, spiders, synapsids, tetrapods, tunicates, and vertebrates.

Literature search was first carried out using all binary combinations of each of the words in the senescence and animal clades sets to identify the general life expectancy patterns of the main groups of animals, the occurrence of senescence symptoms in animals, and the reported causes of death. Next, all binary combinations of each of the words in the animal features and animal clades sets were searched to identify the most important adaptive features of the animal clades.

**Figure 4:**
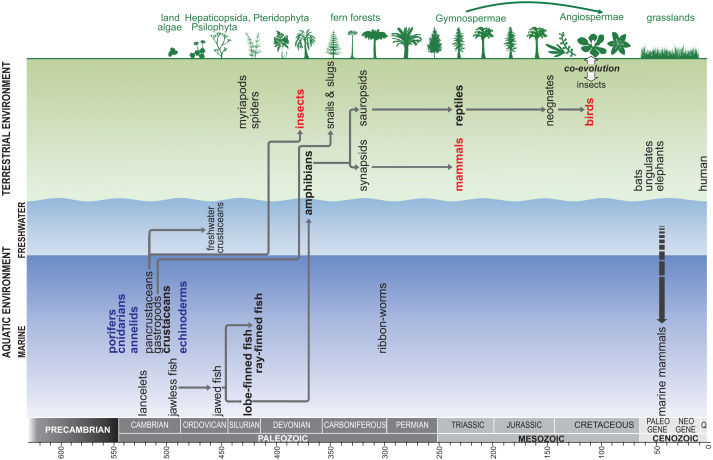
Generalised model showing the phylogenetic relationships between animal clades, in the context of the major stages of evolution; red –senescent animals, blue –biologically ‘immortal’ animals. Topology is based on the Tree of Life Web Project (http://www.tolweb.org) in addition to many studies from the literature that were used to resolve uncertainty in the ToL project phylogeny.

We then synthesised the data to present a generalised relationship between senescence and life cycles of animals. This may be connected to the ability of cell replacement and regeneration, while considering the biology of the animals and the corresponding environmental factors. Furthermore, we constructed a model showing phylogenetic relationships between animal clades. In the context of the major stages of evolution, we identified the main “senescent” and “biologically immortal” clades of animals. Topology was based on the Tree of Life (ToL) Web Project (http://www.tolweb.org) in addition to many studies from the literature that were used to resolve uncertainty in the ToL project phylogeny (Fig. 4).

Finally, we juxtapose our synthesis of the literature review with the most important concepts and theories explaining the origin and mechanisms of senescence. We present our conclusions and hypotheses that provide an explanation of the causes of senescence symptoms and attempt to indicate their genesis in the main clades of terrestrial animals and the relationship with life expectancy and maximal lifespan.

We discuss mainly three clearly senescing groups of terrestrial animals, that is, insects, birds, and mammals. This choice was based on their dominance in terrestrial environments. Hence, we tried to address the following questions: Why do these three clades, which are so successful on land and yet so different, show symptoms of senescence? Could senescence be adaptive? In addition, can the secondary aquatic reptiles and mammals live longer than their relatives that have remained on land?

### Animals with different degrees of senescence

No evolutionary mechanisms propose the programmed death of the individual; instead, senescence is thought to be a consequence of reduction the effectiveness of natural selection with age (see ‘the selection shadow theory’ (Flatt & Partridge, 2018; Gavrilova et al., 1998; Medawar, 1946). Partridge and Barton advocated that the distinction between parent and offspring is a necessary condition for senescence (Partridge & Barton, 1993).

### Reproduction and growth *vs.* senescence

Animals can be divided into two main groups based on whether they are terminally differentiated in terms of sexual reproduction. The first group contains non-senescent, “biologically immortal” forms (at least in theory, because it can be found papers showing senescence in the asexually reproducing marine oligochaete *Paranais litoralis*, where no distinction between soma and germline (Martínez & Levinton, 1992), which include the simplest animal forms such as cnidarians that reproduce agametically (Reitzel, Stefanik & Finnerty, 2011). The second group contains forms that are irreversibly specialised in sexual reproduction (Sawada, Inoue & Iwano, 2014). Among these animals, some forms (birds) may exhibit symptoms of senescence while others do not (fish). The senescence is observed in the case when an organism switches off the expression of existing growth and regeneration programs (*e.g.*, imago formation in insect development) or when particular programs of growth and regeneration of progenitors are irreversibly lost (*e.g.*, in mammals and birds). In turn, maintaining regenerative capacity prevents or reduce appearance the symptoms of senescence, what can be observed in species, which show ability to continuous growth (Bilinski, Bylak & Zadrag-Tecza, 2016; Bilinski, Bylak & Zadrag-Tecza, 2017). The analysis shows that aging patterns which includes increasing, constant, and decreasing mortality with aging differ between species depending on their ability to determinate or continuous (indeterminate) growth. Species with indeterminate growth, including some animals and most higher plants, seem to escape the senescence, most probably due to their capacity of these species to continuous growth (Jones et al., 2014; Munné-Bosch, 2015). It is connected with Weismman conception of soma and germ-line and disposable soma theory proposed by Kirkwood (Kirkwood & Holliday, 1979). Active program of cell replacement and regeneration allows for the maintenance of the soma and by that occurrence the negligible or negative senescence.

### Symptoms of senescence

Symptoms of senescence include gradual deterioration in function (Niccoli & Partridge, 2012), morphological and physiological changes at cellular (Guerville et al., 2020; Herranz & Gil, 2018; Munoz-Espin & Serrano, 2014) and organismal levels (Dodig, Čepelak & Pavić, 2019), phenotypic changes (McHugh & Gil, 2018; van Deursen, 2014), a reduction in fecundity (Ericsson et al., 2001), and increased risk of age-related mortality (Bilinski, Paszkiewicz & Zadrag-Tecza, 2015).

However, senescence symptoms are not observed in groups of animals such as fish or reptiles, which continue to increase their body size after reaching sexual maturity (Bilinski, Bylak & Zadrag-Tecza, 2016; Speakman, 2005). Although there is some evidence of senescence in fish and reptiles (Gerking, 1957; Shefferson, Mizuta & Hutchings, 2017), negligible senescence phenotypes predominate in these clades (Carey & Judge, 2000; Froese & Pauly, 2019; Woodward, Horner & Farlow, 2011). In turn, considering the lifespan among vertebrates, fish represent the most numerous species that live over 100 years (Reznick, Ghalambor & Nunney, 2002).

On the other hand, symptoms of senescence are encountered in groups such as birds (Ricklefs, 2010) and mammals (Broussard et al., 2003), in which body growth ceases as soon as the animal reaches sexual maturity. Alternatively, senescence may emerge in insects and related species during the fundamental reconstruction of an organism such as during complete metamorphosis (Abdelwahab et al., 2018; Nöthiger, 1972).

### Regenerative potential *vs.* senescence

Continuous growth is accompanied by high regeneration and cell replacement capabilities (Bilinski, Bylak & Zadrag-Tecza, 2016). However, even if animals do not grow continuously, some species retain the ability for continuous growth and regeneration in individual parts of the body (Alvarado, 2000). For example, female tarantulas moult throughout their whole life (Costa & Perez-Miles, 2002) and some parts of the human body such as ears, grow continuously (Niemitz, Nibbrig & Zacher, 2008). The possibility of continuous growth reduces the occurrence of senescence and promotes longevity. The crucial role of continuous growth in promoting longevity is best manifested in extremely long-lived species such as the giant tortoise (*Aldabrachelys gigantea*) (Quesada et al., 2019), orange roughy (*Hoplostethus islandicus*) (Andrews, Tracey & Dunn, 2009), and Greenland shark (*Somniosus microcephalus*) (Nielsen et al., 2016). In other long-lived forms, such as termite queens, substantial increases in body size are observed in imagos, a rather unique phenomenon among eusocial species of insects (Heinze & Schrempf, 2008; Keller, 1998).

Surprisingly, the most successful clades of terrestrial animals, in terms of total biomass, population size, or numbers of species, are mammals, birds, and insects, which show clear symptoms of senescence (May, 1988; Monaco, Silvestre & Silveira, 2012; Mora et al., 2011). Hence, senescence coincides with the ability of animal clades to adapt to a wide variety of terrestrial habitats.

### Main physical factors hindering the colonisation of land by animals

As a frame for the paper, we explore the evolutionary constraints imposed by colonization, which may help explain the onset of senescence. We try to not develop the side threads and avoid the intricacies of the mortality question. We begin with a brief overview of the animal adaptations needed to make the transition from the aquatic environment to the terrestrial environment.

### Respiratory system

When invading land, animals had to overcome various constraints (Bray, 1985) such as supplying tissues with oxygen and removing carbon dioxide. Gills were useless on land as they dried out easily (Hsia et al., 2013). This problem was solved in insects through the direct supply of air to tissues by the system of capillary structures (trachea).

As insects increased in size, this oxygen supply system became less efficient (Nation, 2005), making a significant increase in the body size of insects nearly impossible (Hanken & Wake, 1993; Polilov, 2016). Thus, the miniaturisation of body size (Table 1), frequently observed in insects, significantly improved oxygen supply (Molnar & Gair, 2015; Nation, 2005). Accordingly, the maximum body size of insects (Nation, 2005) is strongly dependent on the oxygen concentration in the air (Burggren & Infantino, 1994; Whitford, 1973). Therefore, insects could grow to large sizes when the oxygen concentration in the air reached 38% in the Carboniferous and Permian ages (Harrison, Kaiser & Vanden Brooks, 2010; Molnar & Gair, 2015).

**Table 1:** Life histories of insects *vs*. possible factors causing the loss of continuous growth and regeneration, and the onset of senescence. Cat. –Generalized thematic categories.

Cat.	Discussion of important features and events
Ancestors of insects	• Insects are the senescent clade which evolved from a probably non-senescent group of Pancrustaceans (Tamone & Harrison, 2015).
Respiratory system	• The small body size of insects is the consequence the tracheae serving as the delivery mechanism for oxygen (Hanken & Wake, 1993; Polilov, 2016).
Short adult life	• The short life of sexually competent individuals was additionally combined with the lack of feeding ability in the imagoes of some groups of species (Jacobus, Macadam & Sartori, 2019).
	• This limitation was additionally reinforced by the fact that role of the insect imago is to lay many fertilised eggs (Berger, Walters & Gotthard, 2008) after efficiently dispersing, enabled by its flight capability (Alves et al., 2019; Compton et al., 2002).
	• After laying the eggs, the fate of the imago is no longer important (Rosenheim et al., 2008).
Importance of metamorphosis	• The complete and irreversible metamorphosis of insects results in a drastic simplification of life processes, effectively limiting the imago to a one-time only participation in the reproductive process (Rosenheim et al., 2008; McMahon & Hayward, 2016; Rolff, Johnston & Reynolds, 2019).
	• The efficiency of this reproductive strategy has ensured the evolutionary success of this group of animals (Rolff, Johnston & Reynolds, 2019).
	• The inactive and therefore vulnerable pupa stage is shortened as much as possible (Lindstedt, Murphy & Mappes, 2019).
Possibility of regeneration	• The formation of the imago body from a number of separate parts of imaginal discs resembles the production and final assembly of parts rather than the stepwise development of the body in the larvae. For the sake of speed and large-scale production characteristics for that group, “products” are disposable (Nöthiger, 1972).
	• A high vulnerability to mechanical damage of their wings, which are made mostly from “dead” cells (cuticle) (Pass, 2018), makes any repair virtually impossible. Consequently, regeneration/repair mechanisms known in the larval stages for other parts of the body became inefficient and eventually disappeared in imagoes (Abdelwahab et al., 2018).
	• As in the case of disposable products in contemporary industries, sexually competent stages of insects cannot repair any broken parts of the body as the information on the mechanisms for repair is no longer available after a complete metamorphosis (Parle, Dirks & Taylor, 2016).

The first amphibians used the circulatory system already existing in fish (Snyder & Sheafor, 1999), modified by the formation of structures for gas exchange (lungs) (Thomson & Bossy, 1970), with haemoglobin as the oxygen carrier. Amphibians, like insects, are highly dependent on the oxygen concentration in the air, and their maximum sizes were also found in the Carboniferous and Permian hyperoxia periods (Harrison, Kaiser & Vanden Brooks, 2010). Some animals exchange gases not only through internal systems but also directly through the skin (Brainerd, 1999), and some species of amphibians have no lungs (Hutchison, 2008). Since increasing activity on land required greater efficiency of the respiratory system, reptiles underwent a substantial reconstruction (Garwood & Edgecombe, 2011; Little, 2009). Similarly, the air sac system and breathing movements evolved in birds (Hanken & Wake, 1993; Maina, 2015; Polilov, 2016).

### Problem of desiccation and movement

The impact of other adverse physical factors, such as UV irradiation and desiccation (Hessen, 2008), was much easier to resolve. For instance, the presence of gas impermeable cuticles (insects) (Moussian, 2010) or dry scaly skin (reptiles) (Rutland, Cigler & Kubale Dvojmoč, 2019) prevented desiccation. However, the problem of movement was the most difficult to solve (Padian, 2016). Insects evolved from *Pancrustaceans* that had hard, crusty exoskeletons and locomotory limbs (Tamone & Harrison, 2015), which were adapted for both swimming and walking, and therefore, could be easily implemented on land (Rolfe, 1985). Terrestrial insects also developed flight capabilities, making them more dispersible than their arthropod or mollusc competitors (Yanoviak, Kaspari & Dudley, 2009). The problem of movement in tetrapods was solved later by the evolution of fins with a fleshy base in lobe-finned fishes (*Sarcopterygii*). Fins that were previously used for swimming were thus transformed into limbs for walking on land (Little, 2009; Long & Gordon, 2004; Oster et al., 1988). The flight ability of terrestrial vertebrates (Tables 2 and 3) appeared much later than that of insects (Garwood & Edgecombe, 2011; Hunter, 2007).

**Table 2:** Life histories of birds *vs.* possible factors causing the loss of continuous growth and regeneration, and the onset of senescence. Cat.– Generalized thematic categories.

Cat.	Discussion of important features and events
Ancestors of birds	• Birds originated from probably continuously growing dinosaurs (Little, 2009).
	• Based on the structure of the bones of dinosaurs, some authors drew conclusions about the existence of senescence in this group of animals (Woodward et al., 2015).
	• However, other research carried out on alligators, which are representatives of modern sauropsids from which birds also emerged, showed that alligators do not senesce (Woodward, Horner & Farlow, 2011).
Adaptations to flight	• Adaptations to the environment developed in the course of evolution of terrestrial vertebrates could also contribute to the limitation of regenerative abilities and strong limitation of growth in sexually mature adults, favouring the onset of senescence.
	•The ability of flight (Dudley et al., 2007) facilitated bird access to resources, dispersion, and ability to avoid predators.
	• Overcoming the effects of gravity gave birds a leading role among the terrestrial tetrapods, in terms of migration distance. Birds are the only terrestrial vertebrates that share with humans the peculiarity of traveling in a few hours across intercontinental borders (Jourdain et al., 2007).
	•Flight in most of contemporary birds, is based on frequent wing beats, which requires a high rate of metabolism (Bairlein et al., 2015; Roots, 2006).
	• The additional evolution of mechanisms that increased the gas exchange rate was necessary, and thus, air sacs were developed (Puttick, Thomas & Benton, 2014).
Types of flight	• The first type of flight is based on frequent wing beats (small birds; passerine-type flight sensu (Bruderer et al., 2010) and requires a high energy expenditure (Heers et al., 2016; Savile, 1957).
	• The other type of flight combines gliding and soaring and has a much lower energy requirement; however, it requires a much larger wingspan and a lower proportion of body weight to the surface of the wings (Maina, 2000), just like vultures soaring over land (Newman, 1958; Pennycuick, 1973) and albatrosses on the sea (Meyers & Stakebake, 2005) are the other extreme.
	• In moderately sized birds, active flight dominates and is accompanied by periods of gliding, as in the case of Galliformes (Andrews, Mackenzie & Gregory, 2009; Heers et al., 2016; Meyers & Stakebake, 2005; Savile, 1957; Andrews, Mackenzie & Gregory, 2009).
	• Because of physical characteristics, birds cannot change from one type of flight (i.e., passerine-type flight) to another (i.e., soaring and gliding) (Vincze et al., 2019).
Relationship of body size with the ability to fly	• The thickness and structure of bird bones are a compromise between physical strength and the requirement for lightness (Dumont, 2010).
	• The physical factor that prevents an alteration in the type of flight for birds is the density of the air (Altshuler & Dudley, 2006; Schmaljohann & Liechti, 2009).
	• A continuous body size increase in birds would create the need to change the mechanisms of flight during their life cycle, which would be an additional problem. Birds that are small at the beginning of their life would need the active flight mechanisms, which could be disastrous for the durability of their wing bones when their size increases (Schmaljohann & Liechti, 2009).
	• Transitioning from the active form of flight to soaring would not be easy to implement during continuous growth (Hill, Wyse & Anderson, 2012).
	• In birds, the increase in the body size made flight mechanically difficult, which led to the evolution of flightless species (Blanckenhorn, 2000; McNab, 1994).

### Temperature in the environment

Adapting to temperature fluctuations in the terrestrial environment is another major challenge. While the temperature of marine waters is relatively stable, temperature varies dramatically in terrestrial environments according to geographic location, depth, ocean currents, and seasons (Archer et al., 2004). Moreover, variation in temperature from below the freezing point to the values at which proteins become denatured (Bischof & He, 2006) is common in terrestrial habitats. Therefore, animals living under extreme temperatures had to develop appropriate protective molecular and behavioural solutions to survive (Angelini & Ghiara, 1984). Accordingly, the activity of ectotherms depends on external temperatures at moderate temperatures, while they are mostly inactive during cold seasons (Costanzo & Lee, 2013). In contrast, endothermy, which is seen in extant birds and mammals, assures constant temperatures under a broad range of air temperature values, enabling continuous activity (Hill, Wyse & Anderson, 2012).

Thus, the transition to land and the subsequent evolution of insects, birds, and mammals was costly. We propose that these costs led to constraints, and the key constraint was the cessation of growth at maturity. This cessation of growth may have, in turn, led to senescence.

### Senescent animals dominate in terrestrial environments

Senescence may have appeared in animals over the course of evolutionary changes associated with key adaptations to life on land. Therefore, the relationship between successful land colonisation and senescence merits further analysis. Tables 1–3 present the factors that may have contributed to the loss of the ability for indeterminate growth and the reduction/loss of the potential for regeneration in adults, and thus, the appearance of senescence. We must examine the physical aspects of the environment (Hackett et al., 2008) to explain such changes in the life history of these animals.

**Table 3:** Life histories of mammals (including humans) *vs.* possible factors causing the loss of continuous growth and regeneration, and the onset of senescence. Cat. – Generalized thematic categories.

Cat.	Discussion of important features and events
Ancestors of mammals	• Mammals started evolving when terrestrial habitats had already been occupied by insects, other arthropods and molluscs (Gonçalves, 2018).
	• The invasion of land by vertebrates began with amphibian-like creatures, and sometime later, rapidly developing synapsids and sauropsids appeared (Edwards, Paul & Selden, 1992; Kemp, 2006; Pough, Janis & Heiser, 2012).
	• The descendants of sauropsids maintained their dominance among the tetrapods until the end of the dinosaur era. Their extinction made room and provided resources for the synapsids (ancestors of mammals) that had slowly expanded to that point (Oftedal, 2002; Sander et al., 2011).
Early mammals	• During the reign of dinosaurs, early mammals were mainly nocturnal; often burrowing or having secretive habits. Harsh life conditions forced them to develop the mechanism of endothermy accompanied by increases in the levels of their metabolisms and development of insulation (fur) (Gerkema et al., 2013; Luo, 2007).
	• The necessity of hiding in burrows or among rocks during the long time that dinosaurs dominated the landscape could result in slowing down of the continuous growth in body size or in the termination of this growth upon reaching sexual maturity.
	• The development of parental care ultimately led to the appearance of mammary glands and behavioural solutions to protect young progeny until adulthood (Rilling & Young, 2014).
	• A rapid expansion of mammals after the extinction of dinosaurs was the result of partial independence of their activity from external temperatures (Gerkema et al., 2013), which enabled their settlement in climatic zones inaccessible to reptiles (Turbill, Bieber & Ruf, 2011).
Possible limitations of growth	• The appearance of large grassland areas enabled mammals to increase their body size, giving rise to a group of large-sized species (Boyce & Lee, 2010; Cristoffer & Peres, 2003).
	• Numerous habitats supported small or moderate body sizes, including grassland areas where mammals fed on insects, seeds and green parts of grasses and/or roots and tubers, which facilitated burrowing (Little, 2009).
	• However, both large herbivorous mammals and smaller mammal species descended from small ancestors that evolved under heavy pressure from large reptiles. Consequently the earlier, longer lasting pressure to retain a small size resulted in the disappearance of continuous growth ability.

### Regeneration abilities and continuous growth—differences between taxonomic groups

Some groups of aquatic animals, such as certain species of crustaceans, molluscs (Gruber et al., 2015), fish (Nielsen et al., 2016), amphibians (De Magalhaes & Costa, 2009), and reptiles (Cayuela et al., 2019; Sparkman, Arnold & Bronikowski, 2007), do not show clear signs of senescence in contrast to terrestrial taxa, such as insects (Clapham & Karr, 2012; Davies, 1974), terrestrial mammals (Gaillard & Lemaitre, 2017), and birds (Møller, 2007), despite their phylogenetic relatedness. Although the body plans, physiology, and developmental strategies of the three terrestrial groups differ substantially, they have one important feature in common: their sexually mature forms are unable to maintain continuous body growth (Miquel, Economos & Bensch, 1981; Monaco, Silvestre & Silveira, 2012; Moore, 2006).

The loss of continuous growth upon reaching sexual maturity leads to the elimination of characteristic ancestral traits, such as high regeneration ability and the inability to replace worn out somatic cells in imagos of insects, or the limitation of this ability to certain body parts in birds and mammals (Bilinski, Bylak & Zadrag-Tecza, 2016). Hence, only hairs and nails of humans (Lipner & Scher, 2017; Wilson & Tobin, 2010), incisor teeth of rodents (Klevezal, 2010), or eagles’ beaks grow continuously (Galis, Wagner & Jockusch, 2003).

The ability to continue growing after reaching sexual maturity was almost irretrievably lost due to evolution of highly adaptive traits under environmental selection pressures. The convergence of senescence and the ability to colonise terrestrial habitats is consistent with the general theory of trade-offs (Stearns, 1992). The loss of growth is often accompanied by a loss of regenerative capacity.

Insects, as imagos, are incapable of continuous growth and do not exhibit regeneration. Adaptation of insects to the terrestrial environment could have favoured the emergence of senescence in this clade. Insects seem to be the best (and rare) examples of the “disposable soma” theory (Kirkwood, 1977). The loss of growth and regenerative capacity in adult insects appears to be due to metamorphosis. Senescence rate varies significantly across birds, mammals, and insects (Jones et al., 2014; Keller & Genoud, 1999). However, senescent clades such as insects likely evolved from a non-senescent group of *Pancrustaceans* (Tamone & Harrison, 2015), while birds and mammals evolved from sauropsids and synapsids that probably exhibited negligible senescence (Frýdlová et al., 2020). Since sauropsids and synapsids can only be studied on the basis of fossil remains, it is difficult to determine the degree of senescence in these animals, hence the lack of certainty that they did not senescent, but also there is no data confirming with certainty that senescence was visible in them. Woodward, Horner & Farlow (2011) examined the femoral bone microstructure of captive American alligators (*Alligator mississippiensis*) for the presence of an external fundamental system, a form of bone microstructure present in the outermost cortex of long bones in animals that have attained skeletal maturity. The results of this study have important implications for both extinct and extant members of *Sauropsida* (ancestors of birds). Since captive alligators are not senescent, this external fundamental system should not be associated with senescence when interpreting the history of extinct animals such as dinosaurs (Woodward, Horner & Farlow, 2011).

### Maximum life expectancy—differences between taxonomic groups

Our analysis of the maximum life expectancy of species belonging to the groups of animals revealed distinct groups (Figs. 1, 2 and 3). The first group was insects, with a 99% share of short-lived species. However, the maximal lifespan has been significantly extended in some insects, up to several dozen years, but this occurs *via* extension of only the larval phase of life. The second group consisted of amphibians and terrestrial reptiles, with an average maximum life expectancy of about 10 years, though some animals, such as olm (*Proteus anguinus*), tuatara (*Sphenodon punctatus*), and Galapagos tortoise (*Chelonoidis niger*) show extremely high life expectancy. The third group consisted of land mammals and some birds. The average life expectancy of animals in these groups is approximately 15 years. Only humans (*Homo sapiens*) and few other species can be classified as long-lived (Fig. 2). The next group comprised of about 20% long-lived species that live for over 35 years (Fig. 2). This group included fish with short-lived species, but also a large number of very long-lived species, such as Greenland shark (*Somniosus microcephalus*) and deep-water orange roughy. The term Fish is used to refer to the five modern Classes, *i.e., Myxynii* (hagfishes), *Petromyzontida* (lampreys), *Chondrichthyes* (cartilaginous fishes, *e.g.*, chimaeras, sharks, and rays), *Actinopterygii* (ray-finned fishes), *Sarcopterygii* (includes coelacanths, and lungfishes). High average maximum life expectancy was also observed in soaring birds. However, aquatic mammals were completely different from the other groups, with over 40% of them being long-lived (Figs. 1, 2 and 3).

### Senescence as the trade-off between successful land colonization and longevity

#### Terrestrial-marine differences in lifespan

Next, we explored the possibility that senescence, which is quite common in clades of terrestrial animals, might be an adaptive feature. Marine and terrestrial environments differ in terms of their characteristics and requirements for organisms. Marine environments, which enable animals to grow to extremely high body sizes, favoured the capability of indeterminate growth, and therefore, the maintenance of non-senescent phenotypes. These environments are inhabited, in great numbers, by species of primary aquatic vertebrates, such as fish. There is little evidence of senescence in fish (Woodhead, 1998; Woodhead & Ellett, 1966) with some exceptions in species, such as Pacific salmon (*Oncorhynchus spp*.), eels (*Anguilla spp.*), and some lampreys (*e.g.*, *Lampetra planeri*), which reproduce once in a lifetime and die shortly after spawning and laying their eggs (semelparous species) (Finch, 1998; Morbey et al., 2005). In these fish, a single reproductive bout is followed by a rapid physiological decline. The rapid senescence observed in these semelparous fish may be an example of programmed senescence (*via* hormonal cascades), although this is not fully clear (Cohen, 2018).

#### Terrestrial-marine differences in senescence

Terrestrial habitats have been dominated mainly by animals that show a clear senescence phenotype (Carey & Judge, 2000; Welch, 1998), while approximately 93% of angiosperms do not show a senescence phenotype (Baudisch et al., 2013). Therefore, we could infer that there are biological costs of movement. While some sedentary animals, including known non-senescent species such as *Porifera* and *Cnidaria* are present in the aquatic environment, no sedentary animals are found on land. In addition to continuous growth and partial or complete loss of regenerative capacity, locomotion is an ability common to senescent clades of terrestrial animals.

The prevalence of senescing groups of animals in various terrestrial habitats probably results from these species being better able to overcome several physical limitations of this environment. Hence, there may be multiple reasons for mammals and birds to cease their growth (Tables 1–3).

Since the emergence of insects, mammals, and birds occurred at different points of time, senescence must have evolved independently (Fig. 4). Consequently, senescence in these groups of animals is likely to be the result of convergent evolution. Thus, we considered the original questions regarding why these three diverse terrestrial clades, show symptoms of senescence and whether senescence could be adaptive. The arguments presented herein suggest that the appearance of senescence in the three major groups of terrestrial animals was a consequence of the evolution of their life histories and as a side effect of the cessation of growth in sexually mature adults. The main mechanisms leading to the loss of this ability for growth were different across clades and occurred at distinct periods of time. The appearance of senescence in various unrelated clades during various periods of animal evolution suggests convergent evolution of senescence, and hence, a lack of homology.

The relationship between the evolution of senescence and the ability to populate terrestrial habitats requires explanation. In our earlier studies (Bilinski, Bylak & Zadrag-Tecza, 2016; Bilinski, Bylak & Zadrag-Tecza, 2017), we postulated that senescence and “unavoidable” mortality for most groups of animals are not genuine traits but side effects of the evolution of other important traits (known as spandrels) (Gould, 1997; Gould & Lewontin, 1979). Therefore, senescence is not an adaptive trait (Fig. 2). The loss of the ability to continue growth after reaching sexual maturity may result from the earlier evolution of highly adaptive traits.

#### Stable environmental conditions *vs.* senescence

Evidence that the loss of ability of indeterminate growth in terrestrial mammals results in senescence, is provided by secondarily aquatic mammals (Table 3, Figs. 2–3). In addition to lower rate of senescence, tetrapods that have undergone secondary aquatic adaptation include the longest-living mammals (Thewissen et al., 2009), such as the blue whale (*Balaenoptera musculus*), the fin whale (*B. physalus*), the killer whale (*Orcinus orca*) (Carey & Judge, 2000), and the bowhead whale (*Balaena mysticetus*) (Tarpley & Hillmann, 1998), all of which live for over a hundred. Tarpley and Hillmann examined reproductive materials from mature female bowheads but did not see positive evidence of senescence (Tarpley & Hillmann, 1998). Similarly, the maximum and average lifespan of aquatic and semi-aquatic reptiles, which also secondarily returned to the water, exceed those of their terrestrial relatives. On the other hand, while it is difficult to associate soaring birds with the aquatic environment, these birds live longer than their relatives and fly in a more energy-efficient manner (Fig. 2, and Tables 2, 3). Similarly, certain insects, such as termite queens that live in mounds and provide their own microclimate, splendour beetle larvae (*Buprestis aurulenta*) that hatch and tunnel into the wood with relatively stable thermal conditions, and emerged after 51 years, and periodical cicadas (*Magicicada cassini*) with 17 year-old nymphs that live underground, usually within 61 cm of the surface, feeding on the juices of plant roots (Zeng, 1995) also show relatively long lifespans. Therefore, taking into account both of those traits, the key to solving the problem of longevity, and the presence or absence of senescence may be the choice of an environment that allows less energy expenditure, both for movement and maintaining an appropriate body temperature.

Moreover, when considering mammals that live for over 100 years, we cannot ignore humans. The evolution of an extended lifespan in humans is generally explained by the development of civilisation and, therefore, optimal living conditions (Croft et al., 2015). A similar relationship can be observed in other species of mammals that live significantly longer under optimal conditions, such as zoos, compared to the natural environment (Nussey et al., 2013; Tidière et al., 2016).

## Conclusions

Our analysis suggests that senescence may have emerged as a side effect of the evolution of adaptive features that allowed the colonisation of land. The evolutionary drift into senescent overspecialisation, as evidenced by gigantism or spinescence (loss of teeth and a degenerated form), (Benton, 1989) has been used to explain dinosaur extinction (Archibald, 2012). An analogous situation may apply to modern terrestrial animals. Perhaps specialisation and adaptation of animals to life on land was accompanied by senescent phenotypes, as a side effect of evolution. Thus, senescence in mammals (including humans) may be a trade-off compromise between land colonisation and longevity. In our relatively short synthesis, we presented adaptations that involve animals best suited to life in terrestrial environments. We emphasised that senescence occurred in parallel with highly adaptive traits. Examples of secondary aquatic mammals indicate that it is evolutionarily possible to delay the onset of senescence symptoms. The aquatic environment, which offers conditions that allow animals to grow larger, due to greater density of fluid medium (water) and facilitates the maintenance of appropriate body temperatures (Denny, 2015; Hsia et al., 2013), seems to favour negligible senescent phenotypes or an extremely delayed appearance of the signs of senescence, shortly before the death of the individual (as seen in fish, octopus, and whales). Similarly, the functioning of soaring birds seems to be less costly in terms of energy compared to their relatives with passerine-type flights. Hence, we may conclude that an energy-efficient life in a stable environment can delay the symptoms of senescence and promote a longer life.

Although supported by palaeobiological and ecological examples, our synthesis is only a hypothesis. We hope that further research could confirm this hypothesis or present alternate hypotheses.

**Figure 5:**
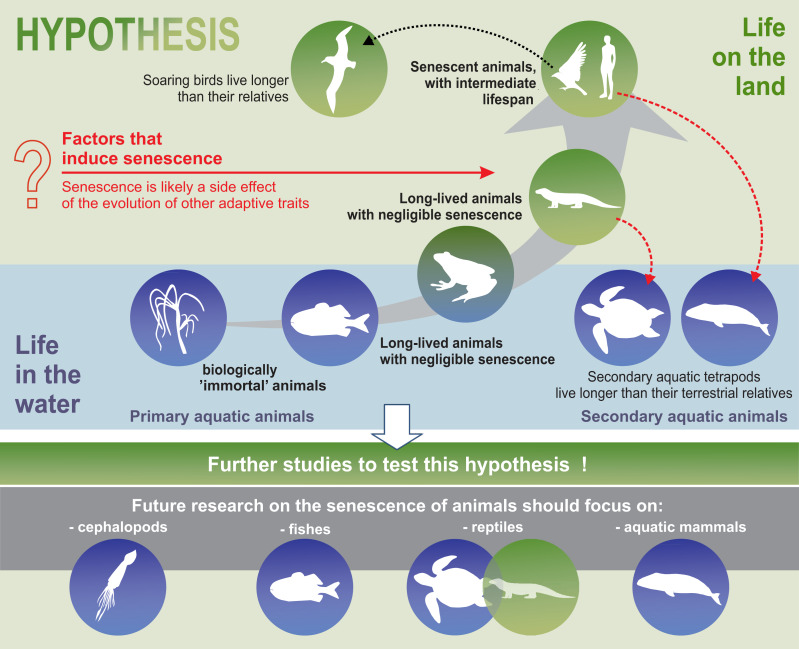
Simplified model of the senescence hypothesis. Model depicting the hypothesis that senescence did not evolve independently but evolved as a side effect of previously chosen developmental strategies.

Furthermore, the hypothesis proposed in this review may constitute the framework for a well-integrated animal biogerontology, combining proximate and future perspectives on the study of senescence. Future research on the senescence of animals should use taxa such as cephalopods, fish, reptiles, and aquatic mammals to test our hypotheses (Fig. 5). Moreover, we believe that, as far as possible, such studies should be conducted in the wild, since patterns of senescence may be distorted in captivity. Senescence should also be separated from individual reactions to adverse environmental conditions. Cephalopods are a good example of the need for separating the external impact, such as the impact of environmental pollution on animals, from the actual symptoms of senescence. Although some experimental studies have been conducted on starvation and memory in the end-of-life phase of these animals (Anderson, Wood & Byrne, 2002), hardly any senescence of cephalopods is observed in nature (Roumbedakis & Guerra, 2019). According to some authors, senescence-like symptoms seen in cephalopods are caused by poor animal welfare (*e.g.*, changes in water quality parameters) (Budelmann, 1998). In light of the many, sometimes dramatic, changes in the aquatic habitats of the world resulting from agriculture, transport, and other industries, undisturbed areas are the most important enclaves for this type of research. In summary, we propose that an energy-efficient life under stable environmental conditions can delay senescence and suggest that future studies attempt to test this hypothesis.

##  Supplemental Information

10.7717/peerj.12286/supp-1 Supplemental Information 1 Summary of the main life patterns of animals in clades

10.7717/peerj.12286/supp-2 Supplemental Information 2 Summary table of reviewed literature

## Additional Information and Declarations

### Data Availability

The following information was supplied regarding data availability: This article is based on the published literature data and the data available in the database, which was reported in the references and “Survey methodology” section.
