# Supplementary material for: Senescence as a trade-off between successful land colonisation and longevity: critical review and analysis of a hypothesis
Source: PeerJ. 2021 Nov 2;9:e12286. doi: 10.7717/peerj.12286 (PMC8570163; doi:10.7717/peerj.12286)
Supplement: Supplemental Information 1 [file peerj-09-12286-s001.docx]

**Senescence as a trade-off between successful land colonisation and longevity: Critical review and analysis of a hypothesis**

Tomasz Bilinski^1^, Aneta Bylak^2^, Krzysztof Kukuła^2^ and Renata Zadrag-Tecza^3^

^1^Department of Biochemistry and Cell Biology, Faculty of Biology and Agriculture, University of Rzeszów, Rzeszów, Poland

^2^Department of Ecology and Environmental Protection; Institute of Agricultural Sciences, Land Management and Environmental Protection, University of Rzeszów, Rzeszów, Poland

^3^Department of Biochemistry and Cell Biology, Institute of Biology and Biotechnology, University of Rzeszów, Rzeszów, Poland

Corresponding Author:

Renata Zadrag-Tecza

Zelwerowicza 4, 35-601 Rzeszów, Poland

Email address: [retecza@ur.edu.pl](mailto:retecza@ur.edu.pl)

**Supplementary information**

| **Table S1**. Summary table of the main life patterns of animals in clades; *- Classes: Chondrichthyes, Sarcopterygii and Actinopterygii; **- An animal capable of completely reverting to a sexually immature stage having reached sexual maturity (Bavestrello, Sommer & Sarà, 1992). | | | | |
| --- | --- | --- | --- | --- |
| **Clades** | **Main life patterns** | | | |
|  | **Type of senescence** | **Lifespan (years)** | **Lifespan category** | **Longevity records (years)** |
| Annelida | biologically ‘immortal’ | 2-1000 | - | *Escarpia laminate* |
| Aschelminthes | senescent | 1-10 | short | *Ascaris lumbricoides* |
| Bivalvia | negligible senescence | 10-507 | long | *Arctica islandica* |
| Bryozoa | not defined | 1-86 | intermediate | *Celleporaria fusca* |
| Cephalopoda | negligible senescence? | 1-25 | intermediate | *Nautilus pompilius* |
| Chelicerata | not defined | 0.02-43 | intermediate | *Gaius villosus* |
| Cnidaria | biologically ‘immortal’ | 5-4265-? | - | *Leiopathes glaberrima*  *Turritopsis nutricula*** |
| Crustaea | negligible senescence | 2-140 | long | *Homarus americanus* |
| Echinodermata | biologically ‘immortal’ | 8-200 | - | *Strongylocentrotus franciscanus* |
| Gastropoda | negligible senescence | 1-40 | long |  |
| Insecta | senescent | 0.06-50 | short | Isoptera - queen |
| Myriopoda | not defined | 4-10 | intermediate | *Archispirostreptus gigas* |
| Platyheminthes | biologically ‘immortal’ | 0.18-3 | - | not defined |
| Porifera | biologically ‘immortal’ | 2300-15000 | - | *Scolymastra joubini* |
| Rotifera | senescent | 0.02-0.125 | short | not defined |
| Cephalochordata | not defined | 4-8 | intermediate | *Branchiostoms lanceolatum* |
| Tunicata | not defined | 1-3 | short | *Botrylloides* |
| Myxini | negligible senescence | 17-40 | intermediate | *Eptatretus stoutii* |
| Cephalaspidomorpha | negligible senescence | 4-10 | intermediate | *Lampetra tridentata* |
| Pisces* | negligible senescence | 1-512 | long | *Somniosus microcephalus* |
| Amphibia | negligible senescence | 1.7-102 | intermediate | *Proteus anguinus* |
| Reptilia | negligible senescence | 1-200 | intermediate/long | *Sphenodon punctatus* |
| Aves | senescent | 1.5-70 | intermediate/long | *Bucorvus cafer* |
| Mammalia -terrestrial | senescent | 0.5-122.5 | intermediate | *Homo sapiens* |
| Mammalia -aquatic | senescent | 13-211 | long | *Balaena mysticetus* |
